# Body weight and body surface area of adult patients with selected cancers: An Italian multicenter study

**DOI:** 10.1371/journal.pone.0314452

**Published:** 2024-12-17

**Authors:** Valentina Danesi, Alice Andalò, Martina Cavallucci, William Balzi, Nicola Gentili, Mattia Altini, Roberta Maltoni, Ilaria Massa, Giorgia Vallicelli, Maria Teresa Montella, Carla Masini, Andrea Roncadori

**Affiliations:** 1 Outcome Research, Healthcare Administration, IRCCS Istituto Romagnolo per lo Studio dei Tumouri (IRST) “Dino Amadori”, Meldola, Italy; 2 Data Unit, Healthcare Administration, IRCCS Istituto Scientifico Romagnolo per lo Studio Tumouri (IRST) “Dino Amadori”, Meldola, Italy; 3 Assistenza Ospedaliera Regione Emilia-Romagna, Bologna, Italy; 4 Unità Operativa Ricerca Valutativa e Policy dei Servizi Sanitari, Ausl della Romagna, Ravenna, Italy; 5 Healthcare Administration, IRCCS Istituto Scientifico Romagnolo per lo Studio Tumouri (IRST) “Dino Amadori”, Meldola, Italy; 6 Oncological Pharmacy Unit, IRCCS Istituto Scientifico Romagnolo per lo Studio Tumouri (IRST) “Dino Amadori”, Meldola, Italy; Nelson Mandela African Institute of Science and Technology, UNITED REPUBLIC OF TANZANIA

## Abstract

Although body weight (BW) and body surface area (BSA) are utilized to establish the appropriate dosage of anticancer drugs, their distribution in cancer patients is poorly studied, making it challenging to predict the amount of drug use and related costs of BW or BAS-dosed regimens. This study investigates the distribution of BW and BSA in adults with selected cancers who initiated systemic anticancer treatment in the eastern Emilia-Romagna region hospitals between 2011 and 2021. BW and BSA were collected at the first cycle of each new treatment line, with multiple measurements for patients receiving various treatments or treating for other primary malignancies. Results were grouped by sex, tumor site and treatment setting, and the normal distribution hypothesis was tested for each group. Both linear regression model and quantile regression at the 50^th^, 25^th^ and 75^th^ percentiles were run to explore the factors influencing BSA. The analysis included 20,634 treatment lines and the corresponding BW and BSA measures from a sample of 13,036 patients. The average BW was 68.05kg (64.20kg for females and 75.07kg for males) and the average BSA was 1.76m^2^ (1.66m^2^ for females and 1.87m^2^ for males). In women, the highest BW was in breast and colon groups, while in men, it was associated with prostate and rectum cancers. The model indicated significant association between BSA, age, sex and tumor localization. Notably, stomach and lung cancers were linked to lower BSA for both sexes (for females -0.081 and -0.041m^2^ respectively compared to those with breast cancer). Advanced settings were related to lower BSA than neoadjuvant treatment, especially for stomach cancer patients, who experienced a weight loss of 3 to 6kg as therapy progressed. The regression models for predicting BSA can assist regulatory bodies in determining reimbursement for new chemotherapy drugs and help hospitals forecast drug utilization and expenditure more accurately.

## Introduction

In clinical oncology, body weight (BW) and body surface area (BSA) are traditionally used to calculate the dose of individual patients’ anticancer regimens. BSA is used to estimate optimal dosage to guarantee drug efficacy, reduce drug toxicity, and account for some changes in pharmacokinetics depending on patient factors. In cancer patients, BW can fluctuate due to various factors, such as treatment side effects, changes in appetite, and disease progression [1–6]. It is crucial to monitor BW throughout treatment as weight changes can affect treatment efficacy and may indicate the need for adjustments in treatment plans [7]. All BSA formulas include patients’ weight and height [8–11]. The most commonly used equations for determining BSA are Du Bois and the Moisteller formula [8, 11]. Distributions of BW, BSA or body mass index (BMI) have been documented from nationally representative samples, sometimes focusing on the changing trend in BW over time and the prevalence of overweight [12–14] or associated with the onset of cardiovascular disease and diabetes [15, 16]. However, despite the widespread use of BW and BSA in oncology, limited data on their distributions in cancer patient populations are available in the literature, none specific to the Italian population. A mean BSA of 1.73 m^2^ was estimated by the author Ratain [17] and resulted from the European Organization for Research and Treatment of Cancer (EORTC) database for 3000 cancer patients from 1990 to 1998 (unpublished data) [18]. A higher median BSA of 1.86 m^2^ (interquartile range 1.68–2.00 m^2^) was assessed in 1650 adult cancer patients participating in phase I trials from 1991 through 2001 [19]. An Australian study of 2838 patients receiving chemotherapy between 1996 and 2000 estimated an overall mean BSA of 1.80 m^2^ (female 1.70 m^2^, male 1.89 m^2^) [20]. A similar finding came from the study of Smorenburg et al., which reported a mean BSA of 1.80 m^2^ ± 5%, even if the sample considered was only twelve patients [21]. During 2005, there was a mean BSA of 1.79 m^2^ (95% CI: 1.78–1.80) for 3163 adult cancer patients who received chemotherapy in the UK. Among them, the mean BSA for men was 1.91 m^2^ (1.90–1.92) and 1.71 m^2^ for women (1.70–1.72) [22]. Between 2013 and 2014, BSA and BW were measured in 3873 Czech adult cancer patients, consisting of 2476 women and 1397 men. The overall mean BSA was 1.86 m^2^, while the mean BSA of women and men was 1.78 m^2^ and 2.00 m^2^, respectively. The overall mean BW was 76.09 kg, 71.94 kg for women and 83.43 kg for men [23]. It is necessary to update these epidemiological studies, which often conducted on limited samples and in some cases without attention to sex-specific differences.

The US Food and Drug Administration (FDA) recently launched Project Optimus to reform dose optimization in oncology [24]. The FDA suggests assessing pharmacokinetic data to determine whether medication exposure is significantly impacted by age, sex, weight, or race. The Italian national and regional authorities use various reference BSA values for pricing and reimbursement of novel treatments; therefore, standard BSA values are needed for dose and cost calculation. For instance, there needs to be more clarity regarding the value of the standard BSA used by the Italian Medicine Agency (Agenzia Italiana del Farmaco, AIFA) and Veneto Region. The AIFA standard is 1.72 m^2^ for adults (i.e. both males and females) and 1.64 m^2^ for women, whereas the Veneto region standard is 1.80 m^2^ for adults and 1.70 m^2^ for women [25, 26]. Although these values appear similar, little differences can be significant, leading to potential waste and increasing costs. The lack of standards could limit the ability of hospitals, regions and countries to forecast yearly average drug utilization and expenditure of all regimens based on BW or BSA dosing. Additional information on average BSA and BW would result in cost savings, contributing to keeping our healthcare system sustainable. The lack of standards is especially relevant for tumors whose incidence is higher (e.g. colorectal and lung cancer) or sex-specific (e.g. breast and prostate cancer) or involves specific populations (e.g. pediatric patients). This study aims to estimate by sex, tumor site and treatment setting the BW, BSA and BMI distribution of an adult population affected by the most common cancers worldwide in the Emilia-Romagna region’s eastern area (Romagna) between July 2011 and June 2021.

## Methods

A multicenter cross-sectional and retrospective study was performed to assess the BW, BSA and BMI distributions of oncologic patients who attended Istituto Romagnolo per lo Studio dei Tumori (IRST) or the Local Health Authority AUSL (Azienda-Unità Sanitaria Locale della Romagna, AUSL Romagna) with a catchment area of more than 1 million people. The demographic and clinical datasets were obtained from data registered and maintained in clinical practice in electronic health records and collected for patients who initiated systemic anticancer therapy between the 1^st^ July 2011 and 30^th^ June 2021 to treat breast, colon, lung, rectum, stomach or prostate cancer. Data on sex, tumor(s) type, age and body measurements (BW, BSA, BMI) at the first cycle of each new anticancer treatment and the relative treatment setting (neoadjuvant, adjuvant, advanced first-line, second-line and subsequent lines) were collected. Moreover, all intravenous and oral regimens were retrieved with or without dose calculation. The data extraction took place in December 2021. The authors had no access to information that could identify individual participants during or after collection. Patients’ BW, BSA and BMI were collected at the beginning of each antiblastic regimen whenever a patient started a new treatment for the same primary malignancy (i.e. adjuvant, neoadjuvant, and advanced disease settings as first-line, second-line and subsequent lines) or new regimen(s) for various primary malignancy during the observational period. The database included multiple measures per patient in case the patient underwent different treatment settings or was treated for other tumors [22]. Height and weight were obtained from the database at first drug administration within a line, and BSA was calculated using the Dubois and Dubois method [8]:

$$BSA\left(m^{2}\right)={Weight(kg)}^{0.425}*{Height\left(cm\right)}^{0.725}*0.007184$$ (1)

The BW, BSA and BMI were expressed in kg, m^2^ and kg/m^2^, respectively.

### Ethical approval and informed consent

The study was approved by the Scientific and Medical Committee of IRST and the Romagna Ethical Committee within the Comprehensive Cancer Care and Research Network (CCCRN) project in Romagna, which was set up to form a collaboration between AUSL Romagna and IRST "Dino Amadori" IRCCS aimed at maximizing the activities and the mission of the bodies involved through shared governance of the leading research assets [27]. This study was conducted in accordance with the Declaration of Helsinki. Informed consent was not collected since, according to Italian law, informed consent was unnecessary for retrospective observational studies.

### Study population

Adult patients resident in the Emilia-Romagna region with histologically or cytologically confirmed diagnoses of breast, colorectal, lung, stomach or prostate cancer, who initiated systemic anticancer treatment in Romagna hospitals between July 2011 and June 2021 were included in the study.

### Statistical analysis

Results were clustered by sex, tumor site and treatment setting. A descriptive analysis of BW, BSA and BMI, expressed as percentile scores (1%, 5%, 10%, 25%, 50%, 75%, 90%, 95% and 99%), mean, standard deviation (SD), minimum and maximum values (range) were summarized for each tumor site by sex and treatment setting. The absolute frequencies of the analyzed cases were also reported (N). Shapiro Wilk’s Normality tests were used for assessing the normality distribution of BSA for each relevant subpopulation defined by the combination of sex, tumor location and treatment setting.

Furthermore, in order to explore the possible factors influencing the BSA, a set of statistical models was developed. Specifically, a multiple linear regression model was initially used for estimating the expected values of BSA given a set of covariates (i.e., age, sex, tumor location and treatment setting). Moreover, considering that the BSA distribution was not proven to be normally distributed, quantile regressions at the 50th percentile (i.e., the median) and at the 1st and 3rd quartiles (i.e., 25th and 75th percentiles, respectively) were also run. Indeed, in cases in which the normal distribution of a variable is not demonstrated, quantile regression is a useful tool for assessing the impact of each determinant at different percentiles of the variable distribution. More precisely, being X a vector of observed covariates, the τ^th^ (tau) quantile of BSA conditional on X can be modelled as follows:

$$Q_{yi|xi}\left({\tau |x}_{i}\right)=x_{i}^{T}\beta_{\tau }$$ (2)

Statistical significance was set at p < 0.05. Statistical analyses were performed using R statistical software, version 4.3.1 (www.r-project.org).

## Results

**Fig 1:**
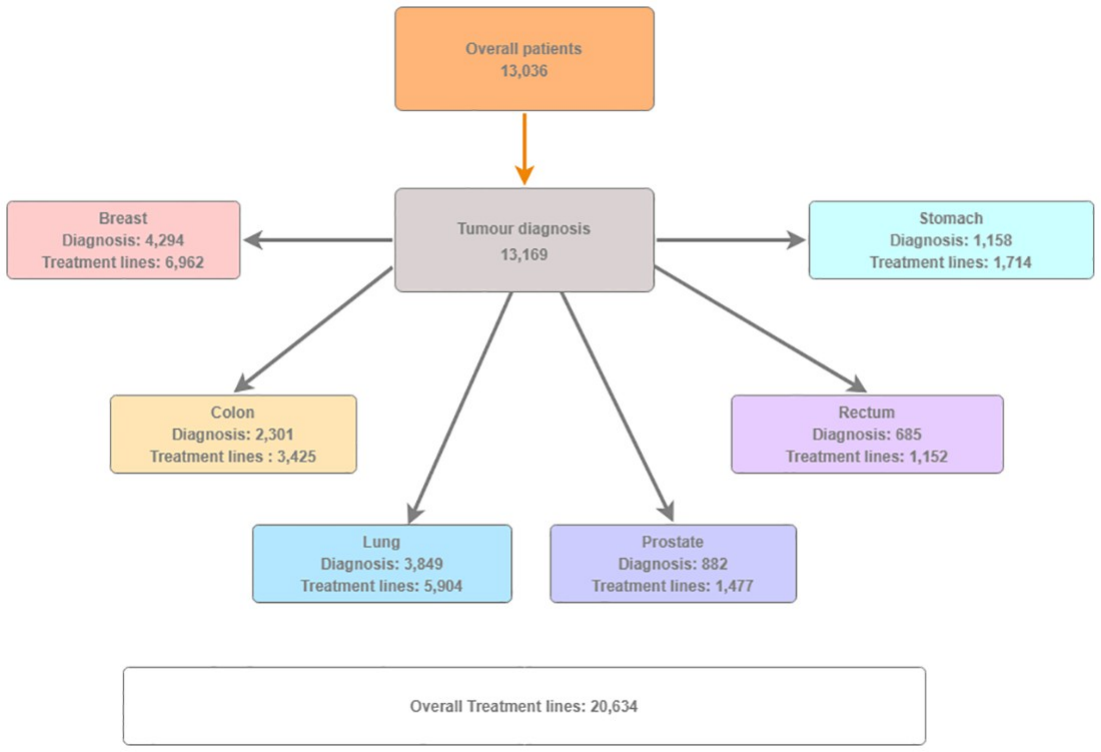
Patients’ disposition. The overall number of patients (13,036) is smaller than the number of diagnoses (13,169), as 123 patients diagnosed with more than one cancer started anticancer drug therapy for various primary cancers during the observational period. The total number of lines of anticancer treatments carried out by patients whose anthropometric characteristics such as BW and BSA were collected at each beginning of the new treatment was 20,634. The database included multiple measures per patient in case the patient underwent various treatment settings for same cancer or was treated for multiple tumors.

The analysis included 13,036 patients diagnosed with 13,169 primary cancers (Fig 1). The sample had a median age of 67 years (IQ range: 57–74). Female patients were 56.05% with a median age of 63 years (IQ range: 53–72), whereas male patients accounted for 43.95% with a median age of 70 years (IQ range: 63–77). 13,169 were the number of primary cancer diagnoses established in the sample, which included 123 patients with multiple primary cancers. Over half of the patients were diagnosed with breast cancer (32.61%) and lung cancer (29.23%), whereas rectal cancer was the least common diagnosis (4.49%) (Fig 1). The total number of new treatment lines was 20,634 (Fig 1), which matched the number of BW and BSA measurements performed at each initiation of anticancer drug therapy. More than half of the treatment lines were carried out by women (N = 11614, 56.28%).

The overall mean BW in the whole population was 68.05 kg. When considering the population according to their sex separately, a mean BW of 64.20 kg and 75.07 kg was observed in females and males, respectively (Table 1). Among females, the highest average BW was measured in patients with breast cancer (65.78 kg). Rather, patients with prostate cancer (78.88 kg) and rectum cancer (77.45 kg) had the highest average BW in males. Conversely, gastric cancers were shown to have the lowest BW in both sexes (Table 1).

**Table 1:** Cancer patients’ BW distribution.

Sex	Tumor site	N	1%	5%	10%	25%	50%	75%	90%	95%	99%	Mean	SD***	Range (Min-Max)
F*	Breast	6,962	45	49	51	57	64	73	82	90	103	66	13	40–135
	Colon	1,517	41	45	48	54	61	70	80	85	102	63	13	40–140
	Lung	2,168	40	45	47	52	60	69	77	85	100	62	12	40–140
	Rectum	376	40	45	48	52	60	70	80	85	100	63	13	40–117
	Stomach	591	40	42	45	50	58	65	75	82	96	59	12	40–118
	**Overall females**	**11,614**	**42**	**46**	**50**	**55**	**62**	**70**	**80**	**88**	**102**	**64**	**13**	**40–140**
M**	Colon	1,908	50	57	60	67	75	83	93	100	118	76	13	44–132
	Lung	3,736	49	55	59	65	73	80	90	97	110	74	13	40–172
	Prostate	1,477	51	60	63	70	78	87	97	104	120	79	14	44–140
	Rectum	776	50	58	61	68	76	85	95	100	120	77	14	46–175
	Stomach	1,123	47	53	56	62	70	79	89	95	113	71	13	40–135
	**Overall males**	**9,020**	**50**	**55**	**60**	**65**	**74**	**82**	**92**	**100**	**116**	**75**	**14**	**40–175**
	**Overall**	**20,634**	**43**	**49**	**52**	**59**	**68**	**78**	**88**	**95**	**110**	**69**	**14**	**40–175**

*Females

**Males

***Standard Deviation

The BW (kg) measures collected at the beginning of each treatment line were split according to the sex (F and M) and tumor site. Lung included both NSCLC (non-small cell lung cancer) and SCLC (small cell lung cancer). The third column (N) reports the number of new treatment lines initiated, corresponding to the number of BW measures performed at the beginning of a new treatment. Data were expressed in the nth percentile (1%, 5%, 10%, 25%, 50%, 75%, 90%, 95% and 99%), mean, standard deviation (SD) and minimum and maximum value (Min-Max).

A similar trend was observed in analyzing BSA (Table 2) and BMI data (S1 Table). The overall mean BSA was 1.75 m^2^, corresponding to 1.66 m^2^ in females and 1.87 m^2^ in males.

**Table 2:** BSA distribution.

Sex	Tumor site	N	1%	5%	10%	25%	50%	75%	90%	95%	99%	Mean	SD***	Range (Min-Max)
F*	Breast	6,962	1.38	1.45	1.50	1.58	1.68	1.78	1.89	1.96	2.102	1.69	0.15	1.25–2.33
	Colon	1,517	1.35	1.40	1.45	1.53	1.63	1.74	1.85	1.92	2.113	1.65	0.16	1.30–2.32
	Lung	2,168	1.33	1.40	1.43	1.52	1.62	1.73	1.84	1.91	2.088	1.63	0.16	1.27–2.27
	Rectum	376	1.31	1.40	1.43	1.50	1.61	1.76	1.86	1.97	2.119	1.63	0.18	1.29–2.23
	Stomach	591	1.31	1.36	1.40	1.49	1.58	1.69	1.79	1.86	1.975	1.60	0.15	1.27–2.09
	**Overall females**	**11,614**	**1.35**	**1.42**	**1.47**	**1.55**	**1.66**	**1.76**	**1.87**	**1.94**	**2.10**	**1.66**	**0.16**	**1.25–2.33**
M**	Colon	1,908	1.52	1.62	1.68	1.77	1.87	1.99	2.10	2.19	2.32	1.89	0.17	1.39–2.66
	Lung	3,736	1.49	1.59	1.65	1.74	1.85	1.96	2.07	2.15	2.30	1.86	0.17	1.30–2.74
	Prostate	1,477	1.56	1.65	1.70	1.80	1.90	2.02	2.14	2.19	2.32	1.91	0.17	1.39–2.55
	Rectum	776	1.52	1.63	1.69	1.79	1.89	2.01	2.11	2.22	2.42	1.90	0.18	1.41–2.73
	Stomach	1,123	1.48	1.56	1.62	1.71	1.81	1.94	2.06	2.12	2.31	1.83	0.17	1.24–2.54
	**Overall males**	**9,020**	**1.50**	**1.60**	**1.66**	**1.75**	**1.86**	**1.98**	**2.10**	**2.17**	**2.33**	**1.87**	**0.17**	**1.24–2.74**
	**Overall**	**20,634**	**1.37**	**1.45**	**1.51**	**1.62**	**1.74**	**1.88**	**2.01**	**2.09**	**2.25**	**1.75**	**0.19**	**1.24–2.74**

*Females

**Males

***Standard Deviation

The BSA (m^2^) measures collected at the beginning of each treatment line were split according to the sex (F and M) and tumor site. Lung included both NSCLC (non-small cell lung cancer) and SCLC (small cell lung cancer). The third column (N) reports the number of new treatment lines initiated, corresponding to the number of BSA measures performed at the beginning of a new treatment. Data were expressed in the nth percentile (1%, 5%, 10%, 25%, 50%, 75%, 90%, 95% and 99%), mean, standard deviation (SD) and minimum and maximum value (Min-Max).

Patients generally exhibited a slight decrease in weight as treatment advanced (Tables 3–8), except for female patients with lung or colorectal cancer, whose BSA, on the contrary, tended to increase slightly (Tables 4–6). The stomach cancer group showed the most significant weight decrease across treatment pathways (Table 7). Female BSA ranged from 1.62 m^2^ in the neoadjuvant setting to 1.57 m^2^ in the advanced treatment, indicating a loss of roughly 3 kg (Table 7). This weight loss in the men’s group was even more remarkable, reaching about 6 kg (from 1.88 m^2^ to 1.82 m^2^) (Table 6). There was a moderate drop in weight among patients with breast cancer (Table 3). Instead, prostate patients’ weights were more consistently steady (Table 8).

**Table 3:** Breast cancer patients’ BSA (m^2^) distribution by treatment setting.

**Sex**	**Treatment setting/line**	**N**	**1%**	**5%**	**10%**	**25%**	**50%**	**75%**	**90%**	**95%**	**99%**	**Mean**	**SD** **	**Range (Min-Max)**
F*	1. Neoadjuvant	648	1.37	1.47	1.51	1.60	1.69	1.79	1.90	1.96	2.09	1.70	0.15	1.30–2.33
	2. Adjuvant	2,639	1.40	1.47	1.51	1.59	1.68	1.79	1.90	1.97	2.13	1.70	0.15	1.25–2.30
	Advanced—1 line	1,209	1.38	1.45	1.50	1.58	1.68	1.78	1.88	1.96	2.10	1.69	0.16	1.30–2.29
	Advanced—2 line	834	1.37	1.43	1.49	1.57	1.67	1.77	1.88	1.95	2.06	1.68	0.15	1.36–2.19
	Advanced—subsequent lines	1,632	1.36	1.43	1.47	1.55	1.66	1.77	1.87	1.94	2.04	1.66	0.15	1.26–2.18

*Females

**Standard Deviation

The BSA values collected at the beginning of each breast cancer treatment line were split according to treatment setting. The third column (N) reports the number of therapeutic lines for each treatment setting corresponding to the number of BSA measures performed at the beginning of a new treatment. Data were expressed in the nth percentile (1%, 5%, 10%, 25%, 50%, 75%, 90%, 95% and 99%), mean, standard deviation (SD) and minimum and maximum value (Min-Max).

**Table 4:** Lung cancer patients’ BSA (m^2^) distribution by sex and treatment setting.

Sex	Treatment setting/line	N	1%	5%	10%	25%	50%	75%	90%	95%	99%	Mean	SD***	Range (Min-Max)
F*	1. Neoadjuvant	91	1.32	1.37	1.43	1.51	1.62	1.72	1.82	1.86	1.98	1.61	0.15	1.30–1.99
	2. Adjuvant	108	1.37	1.43	1.47	1.53	1.64	1.76	1.85	1.90	2.02	1.65	0.15	1.30–2.03
	Advanced—1 line	1,172	1.33	1.40	1.43	1.51	1.62	1.73	1.84	1.90	2.09	1.63	0.16	1.27–2.27
	Advanced—2 line	492	1.35	1.40	1.44	1.52	1.62	1.73	1.85	1.91	2.09	1.64	0.16	1.30–2.19
	Advanced—subsequent lines	305	1.36	1.39	1.42	1.52	1.64	1.72	1.84	1.94	2.09	1.63	0.16	1.30–2.12
M**	1. Neoadjuvant	197	1.48	1.61	1.67	1.76	1.89	1.99	2.09	2,17	2.32	1.88	0.17	1.46–2.37
	2. Adjuvant	197	1.48	1.61	1.67	1.76	1.89	1.99	2.09	2.17	2.32	1.88	0.17	1.46–2.38
	Advanced—1 line	146	1.54	1.61	1.65	1.76	1.88	1.97	2.11	2.15	2.34	1.88	0.18	1.50–2.74
	Advanced—2 line	2,033	1.49	1.57	1.63	1.73	1.84	1.96	2.06	2.14	2.28	1.85	0.17	1.30–2.68
	Advanced—subsequent lines	889	1.48	1.60	1.66	1.75	1.85	1.96	2.07	2.16	2.31	1.86	0.17	1.38–2.49

*Females

**Males

***Standard Deviation

The BSA values collected at the beginning of each lung cancer treatment line were split according to the sex (F and M) and treatment setting. Lung included both NSCLC (non-small cell lung cancer) and SCLC (small cell lung cancer). The third column (N) reports the number of new treatment lines initiated, corresponding to the number of BSA measures performed at the beginning of a new treatment. Data were expressed in the nth percentile (1%, 5%, 10%, 25%, 50%, 75%, 90%, 95% and 99%), mean, standard deviation (SD) and minimum and maximum value (Min-Max).

**Table 5:** Colon cancer patients’ BSA (m^2^) distribution by sex and treatment setting.

Sex	Treatment setting/line	N	1%	5%	10%	25%	50%	75%	90%	95%	99%	Mean	SD***	Range (Min-Max)
F*	1. Neoadjuvant	11	1.45	1.47	1.50	1.56	1.59	1.66	1.84	1.84	1.84	1.62	0.12	1.44–1.84
	2. Adjuvant	613	1.35	1.41	1.47	1.55	1.64	1.74	1.85	1.93	2.12	1.65	0.16	1.32–2.32
	Advanced—1 line	452	1.36	1.39	1.44	1.53	1.63	1.74	1.85	1.90	2.12	1.64	0.16	1.30–2.20
	Advanced—2 line	229	1.36	1.40	1.43	1.53	1.63	1.74	1.87	1.90	2.11	1.64	0.16	1.35–2.28
	Advanced—subsequent lines	212	1.34	1.37	1.42	1.50	1.62	1.74	1.85	1.90	2.08	1.63	0.17	1.31–2.16
M**	1. Neoadjuvant	21	1.66	1.68	1.70	1.80	1.91	2.05	2.10	2.11	2.25	1.91	0.16	1.65–2.29
	2. Adjuvant	738	1.52	1.63	1.69	1.78	1.87	2.00	2.11	2.19	2.34	1.89	0.17	1.39–2.54
	Advanced—1 line	584	1.50	1.60	1.66	1.76	1.88	1.99	2.10	2.15	2.32	1.88	0.17	1.43–2.43
	Advanced—2 line	290	1.54	1.62	1.66	1.75	1.88	1.98	2.10	2.18	2.28	1.88	0.17	1.50–2.66
	Advanced—subsequent lines	275	1.55	1.64	1.68	1.78	1.87	1.99	2.12	2.19	2.27	1.89	0.17	1.51–2.40

*Females

**Males

***Standard Deviation

The BSA values collected at the beginning of each colon cancer treatment line were split according to the sex (F and M) and treatment setting. The third column (N) reports the number of new treatment lines initiated, corresponding to the number of BSA measures performed at the beginning of a new treatment. Data were expressed in the nth percentile (1%, 5%, 10%, 25%, 50%, 75%, 90%, 95% and 99%), mean, standard deviation (SD) and minimum and maximum value (Min-Max).

**Table 6:** Rectum cancer patients’ BSA (m^2^) distribution by sex and treatment setting.

Sex	Treatment setting/line	N	1%	5%	10%	25%	50%	75%	90%	95%	99%	Mean	SD***	Range (Min-Max)
F*	1. Neoadjuvant	118	1.38	1.41	1.43	1.53	1.63	1.75	1.90	1.98	2.10	1.65	0.18	1.30–2.16
	2. Adjuvant	110	1.34	1.42	1.44	1.48	1.58	1.68	1.83	1.94	2.00	1.61	0.16	1.30–2.06
	Advanced—1 line	80	1.31	1.37	1.42	1.49	1.60	1.79	1.88	1.90	2.10	1.64	0.19	1.29–2.23
	Advanced—2 line	40	1.36	1.41	1.43	1.50	1.66	1.78	1.83	2.02	2.19	1.66	0.20	1.34–2.20
	Advanced—subsequent lines	28	1.32	1.37	1.40	1.55	1.68	1.78	1.81	1.86	1.98	1.66	0.17	1.31–2.01
M**	1. Neoadjuvant	237	1.52	1.66	1.70	1.81	1.91	2.03	2.14	2.23	2.43	1.92	0.18	1.49–2.49
	2. Adjuvant	207	1.54	1.62	1.67	1.77	1.88	1.98	2.11	2.18	2.35	1.89	0.17	1.42–2.48
	Advanced—1 line	155	1.50	1.63	1.67	1.77	1.87	1.98	2.10	2.24	2.44	1.89	0.19	1.41–2.73
	Advanced—2 line	77	1.57	1.65	1.69	1.78	1.89	2.00	2.08	2.26	2.38	1.89	0.19	1.45–2.52
	Advanced—subsequent lines	100	1.59	1.67	1.71	1.78	1.89	2.01	2.13	2.20	2.34	1.91	0.18	1.55–2.66

*Females

**Males

***Standard Deviation

The BSA values collected at the beginning of each rectal cancer treatment line were split according to the sex (F and M) and treatment setting. The third column (N) reports the number of new treatment lines initiated, corresponding to the number of BSA measures performed at the beginning of a new treatment. Data were expressed in the nth percentile (1%, 5%, 10%, 25%, 50%, 75%, 90%, 95% and 99%), mean, standard deviation (SD) and minimum and maximum value (Min-Max).

**Table 7:** Stomach cancer patients’ BSA (m^2^) distribution by sex and treatment setting.

Sex	Treatment setting/line	N	1%	5%	10%	25%	50%	75%	90%	95%	99%	Mean	SD***	Range (Min-Max)
F*	1. Neoadjuvant	87	1.38	1.41	1.46	1.52	1.62	1.70	1.79	1.86	1.93	1.62	0.14	1.38–2.09
	2. Adjuvant	149	1.33	1.40	1.45	1.51	1.59	1.67	1.78	1.88	1.94	1.60	0.14	1.28–2.01
	Advanced—1 line	226	1.31	1.34	1.38	1.47	1.58	1.69	1.81	1.85	2.01	1.59	0.16	1.27–2.06
	Advanced—2 line	82	1.31	1.36	1.38	1.43	1.56	1.69	1.78	1.86	1.94	1.57	0.16	1.27–2.01
	Advanced—subsequent lines	47	1.33	1.36	1.37	1.43	1.58	1.67	1.77	1.87	1.93	1.57	0.16	1.32–1.93
M**	1. Neoadjuvant	150	1.55	1.65	1.68	1.77	1.86	1.96	2.09	2.18	2.38	1.88	0.17	1.53–2.54
	2. Adjuvant	226	1.50	1.55	1.60	1.70	1.80	1.91	2.03	2.07	2.19	1.81	0.16	1.46–2.33
	Advanced—1 line	438	1.44	1.56	1.62	1.70	1.80	1.94	2.06	2.14	2.33	1.82	0.18	1.37–2.48
	Advanced—2 line	201	1.50	1.55	1.61	1.67	1.80	1.92	2.04	2.13	2.27	1.81	0.17	1.42–2.36
	Advanced—subsequent lines	108	1.45	1.53	1.59	1.69	1.84	1.92	2.03	2.09	2.29	1.82	0.18	1.24–2.30

*Females

**Males

***Standard Deviation

The BSA values collected at the beginning of each stomach cancer treatment line were split according to the sex (F and M) and treatment setting. The third column (N) reported the number of new treatment lines initiated, corresponding to the number of BSA measures performed at the beginning of a new treatment. Data were expressed in the nth percentile (1%, 5%, 10%, 25%, 50%, 75%, 90%, 95% and 99%), mean, standard deviation (SD) and minimum and maximum value (Min-Max).

**Table 8:** Prostate cancer patient’s BSA (m^2^) distribution by treatment setting.

Sex	Treatment setting/line	N	1%	5%	10%	25%	50%	75%	90%	95%	99%	Mean	SD**	Range (Min-Max)
M*	Advanced—1 line	760	1.55	1.65	1.71	1.80	1.90	2.02	2.14	2.20	2.35	1.91	0.17	1.43–2.55
	Advanced—2 line	368	1.58	1.65	1.69	1.80	1.91	2.03	2.14	2.23	2.32	1.92	0.17	1.39–2.51
	Advanced—subsequent lines	344	1.58	1.64	1.71	1.80	1.90	2.03	2.12	2.20	2.25	1.91	0.16	1.43–2.43

*Males

**Standard Deviation

The BSA values collected at the beginning of each prostate treatment line were split according to treatment setting. The third column (N) reports the number of therapeutic lines for each treatment setting, corresponding to the number of BSA measures performed at the beginning of a new treatment. Data were expressed in the nth percentile (1%, 5%, 10%, 25%, 50%, 75%, 90%, 95% and 99%), mean, standard deviation (SD) and minimum and maximum value (Min-Max).

After analyzing the various subpopulations based on sex, tumor location, and treatment setting, the normal distribution assumptions were evaluated individually for each of the 28 groups (S2 Table). Surprisingly, the normal distribution hypothesis was rejected infrequently in the considered groups that were examined, and this occurred only in groups with a small sample size, such as male patients with colon cancer in the neoadjuvant treatment setting..

Looking at the regression models developed for assessing the determinants of BSA (Table 9), we observed a high statistical significance for each of the considered factors.

**Table 9:** BSA multivariable linear regression and quantile regression models.

	Ordinary least squares (OLS)	Quantile regression		
	β^a^ (95% CI)^b^	25% (95% CI)^b^	50% (95% CI)^b^	75% (95% CI)^b^
(Intercept)	1.812***	1.666***	1.786***	1.932***
	(1.812,1.812)	(1.666,1.666)	(1.769,1.804)	(1.910,1.953)
Colon F	-0.029***	-0.034***	-0.033***	-0.026***
	(-0.029,-0.029)	(-0.035, -0.033)	(-0.044,-0.023)	(-0.041,-0.011)
Colon M	0.215***	0.205***	0.214***	0.226***
	(0.215,0.215)	(0.205,0.205)	(0.204,0.224)	(0.212,0.239)
Lung F	-0.041***	-0.048***	-0.042***	-0.046***
	(-0.041,-0.041)	(-0.048,-0.048)	(-0.051,-0.033)	(-0.058,-0.034)
Lung M	0.187***	0.180***	0.188***	0.191***
	(0.187,0.187)	(0.180,0.180)	(0.179,0.197)	(0.181,0.201)
Prostate M	0.256***	0.249***	0.253***	0.268***
	(0.256,0.256)	(0.249,0.249)	(0.241,0.266)	(0.253,0.283)
Rectum F	-0.044***	-0.073	-0.060***	-0.022
	(-0.044,-0.044)	(-0.395,0.249)	(-0.084,-0.035)	(-0.053,0.009)
Rectum M	0.224***	0.211***	0.216***	0.230***
	(0.224,0.224)	(0.211,0.211)	(0.199,0.232)	(0.212,0.249)
Stomach F	-0.081***	-0.090***	-0.078***	-0.080***
	(-0.081,-0.081)	(-0.090,-0.090)	(-0.099,-0.057)	(-0.029,-0.029)
Stomach M	0.154***	0.134***	0.148***	0.170***
	(0.154,0.154)	(0.134,0.134)	(0.134,0.162)	(0.154,0.186)
Age	-0.002***	-0.001***	-0.002***	-0.002***
	(-0.002,-0.002)	(-0.001,-0.001)	(-0.002,-0.001)	(-0.003,-0.002)
Adjuvant	-0.012***	-0.015	-0.019***	-0.014**
	(-0.013,-0.012)	(-0.096,-0.066)	(-0.030,-0.009)	(-0.028,-0.001)
Advanced.- 1 line	-0.016***	-0.026	-0.022***	-0.007
	(-0.016,-0.016)	(-0.633,0.581)	(-0.033,-0.011)	(-0.020,0.006)
Advanced.- 2 line	-0.017***	-0.028	-0.021***	-0.010
	(-0.017,-0.016)	(-0.344,-0.288)	(-0.033,-0.010)	(-0.023,0.004)
Advanced.- subsequent lines	-0.023***	-0.036	-0.024***	-0.015**
	(-0.023,-0.023)	(-0.111,0.038)	(-0.035,-0.013)	(-0.029,-0.001)
Statistics	R^2^ = 0.312			
	Adjusted R^2^ = 0.312			
	Residual Std. Error = 0.162			
	F Statistic = 668.000***			

^a^Coefficient

^b^ 95% Confidence Interval

* p-value<0.1;

**p-value<0.05;

***p-value<0.01

**Fig 2:**
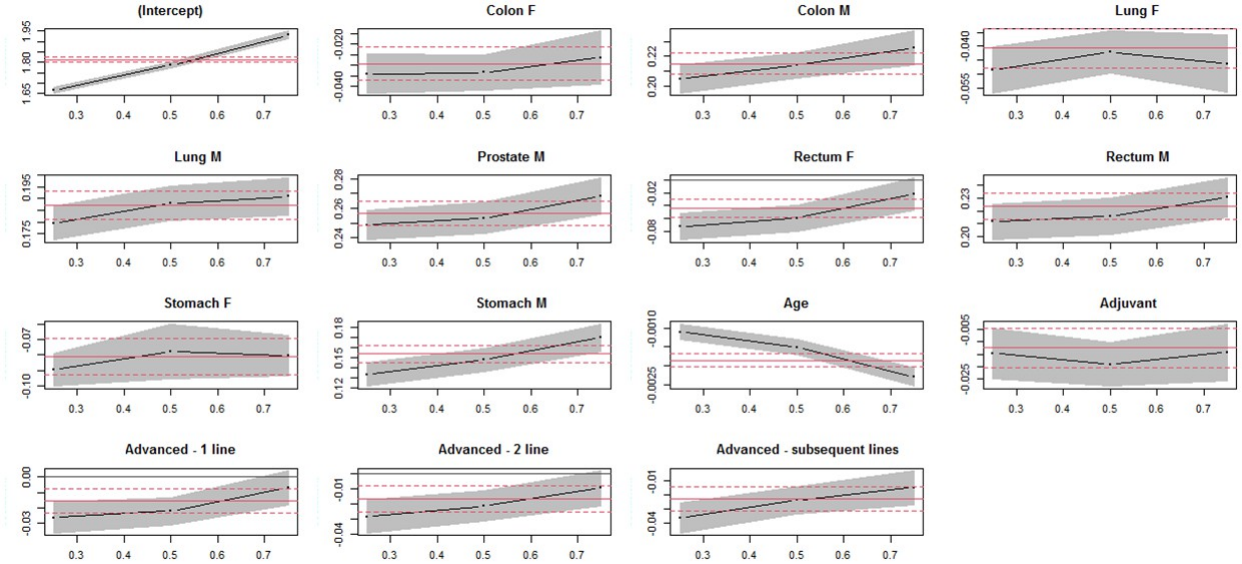
Covariates’ effect on BSA’s quantiles. Plot of covariate effects on quantiles from multivariable quantile regression and their 95% confidence interval (grey area). Note: The parallel to x-axis lines represent the ordinary least squares model estimates and their 95% confidence intervals (solid and dashed lines, respectively).

More precisely, from the multiple linear regression model, compared to a breast cancer patient, gastric tumor localization resulted to be associated with lower BSA for both male and female patients (-0.081 m^2^ for gastric cancer female patients) keeping constant all other factors (i.e., age and treatment setting). Not surprisingly, when adjusting for other covariates, the advanced treatment setting was associated with lower BSA compared to patients undergoing neoadjuvant treatments (-0.016 m^2^, -0.017 m^2^ and -0.023 m^2^ for 1st, 2nd and subsequent advanced lines respectively). Similar results were obtained from the quantile regressions. Notably, the main advantage of using the quantile regression is to evaluate the possible divergence of covariates effect across the BSA distribution percentiles (Fig 2). Indeed, the above described effect of the advanced treatment setting was confirmed only at lower levels of the BSA distribution quartiles (i.e., only at 1st quartile and median), whereas this factor’s effect was not statistically significant at the 75th percentile of BSA.

## Discussion

No standard BW and BSA values for the Italian cancer population are available on which to base accurate drug dose and cost calculations. In this retrospective cross-sectional study conducted in the Emilia-Romagna region (Italy), we found that the average BW of the cancer patients in the eastern area (Romagna) is 68.05 kg. Specifically, females have an average weight of 64.20 kg, while males have an average weight of 75.07 kg. The average BSA in the whole population is 1.76 m2, with females having a BSA of 1.66 m2 and males having a BSA of 1.87. Among individuals of the same sex, variations in BW and BSA are observed across site-specific cancer groups. Overall, most patients experience a limited reduction in BW during therapy, except patients with stomach cancer, who are more susceptible to weight loss and anorexia. Additionally, we have analyzed the BW, BSA and BMI by sex, tumor site and treatment setting. The BSA estimated that sex and overall patient cases are lower than those observed in the British, Australian and Czech cancer populations [20, 22, 23] (S3 Table). Firstly, BW and BSA estimates are affected by the age, sex and tumor site of the sample composition; for instance, in our analysis, more than half of the treatment lines were carried out by women (N = 11614, 56.28%). In addition, this variation could reflect different diets and lifestyles across countries but also changes over time. BW and BSA of a population change in time according to lifestyle, political and economic conditions. As observed by previous studies, BW and BSA tended to decrease with advancing age in both sexes [22]. Differences between particular tumor groups were observed. In particular, the mean BW and BSA for women with breast cancer were higher than those for women with different cancer types, corroborating a previous study [22]. A higher BW and BSA were observed in men with prostate cancer. In both sex groups, the lowest BW and BSA were reported for gastric cancer patients, who are more prone to weight loss and anorexia because of malnutrition [6, 28]. In addition, fluctuations in BW can be frequently observed during the natural history of the disease; therefore, having multiple values of BW from the same patient at the start of therapy can be very informative, allowing to obtain a more balanced measure between the weight at the first line of treatment and the weight at the subsequent lines. Fluctuations in weight are a common occurrence both during and following breast cancer therapy; several studies have reported a gain weight trend, in particular among patients receiving adjuvant chemotherapy in early-stage breast cancer [2, 3, 29, 30]. On the contrary, our findings revealed an opposite trend, showing a weight reduction pattern as shown by Sacco et al. [22]. Studies in the literature demonstrate that high weight in breast cancer is associated with increased morbidity and a worse prognosis [31–33]. For this reason, healthcare professionals are increasingly encouraging lifestyle interventions such as diets and physical activity to achieve weight loss among breast cancer patients. Chemotherapy is known to cause various side effects, including toxicities that can contribute to affecting and changing a patient’s weight. A piece of growing evidence argues that BW and BSA are inadequate to establish the appropriate anticancer drugs dose, suggesting that body composition parameters may better predict chemotherapy toxicities [34]. A previous study on a healthy population living in Italy’s southern and central regions estimated a median BW of 84.3 kg and 68.3 kg for males and females, respectively [35]. According to our findings, women with breast cancer (64.0 kg) and men with prostate cancer (78.0 kg) appear to have BWs most close to those estimated by de Mesquita Barros Almeida Leite and colleagues, although clearly lower. We can assume that the healthy population weighs more than the cancer population, with the gastric cancer population showing the highest differential (negative) compared to the healthy population. However, we do not have enough data to show that our region’s healthy population fully overlaps with that of the central and southern Italian regions.

The quantile regression model developed may be used to estimate the three relevant quartiles (i.e., the median and the 1st and 3rd quartiles) of the BSA in the context of a non-normal distribution to obtain predictions of BSA for the Italian population according to age, sex, tumor location and treatment setting.

To the authors’ knowledge, this study included the most extensive data set on BW, BSA and BMI related to adult cancer patients ever considered before. Furthermore, BSA was assessed across different treatment settings. Unfortunately, ethnicity information was not retrieved. However, most of our sample belonged to “natives” since the regional incidence of foreign is equal to 12.3% [36]. The hospitals in Romagna treat mainly local cancer patients, but their professional and scientific level draws patients from the whole country. In addition, the investigation was carried out in a specific district of our country, so the estimates for BW and BSA may differ from the national average. The main limitations of this study relate to the lack of data on chemotherapy regimens, chemotherapy toxicity, therapeutic agent dose adjustment, and other important factors such as the comorbidities, the nutritional status and diet of patients. Future research should include comparisons among patients receiving curative or palliative chemotherapy for the same diagnosis.

## Conclusion

In summary, attributing average BW and BSA to particular cancer diagnoses would help the national regulatory bodies decide on reimbursement of new therapeutic agents, which for most chemotherapy drugs involves calculations based in part on expected BSA values and improving the forecast of hospitals and regions on drug utilization and waste. Furthermore, the data offer an insight into the anthropometric characteristics of the Romagna population in general and could be used in other fields of medicine. Finally, the developed model may be used to obtain BSA predictions in the Italian populations. Without reliable BW and BSA distribution estimates, these results may be generalized and used for future cost determinations and budgeting for new agents.

## Supporting information

S1 Table Cancer patients’ BMI distribution. (DOCX)

S2 Table Shapiro Wilk Normality test for BW, BSA and BMI distributions. (DOCX)

S3 Table Comparison of mean cancer patients’ BSA among countries. (DOCX)
