# Supplementary material for: Body weight and body surface area of adult patients with selected cancers: An Italian multicenter study
Source: PLoS One. 2024 Dec 17;19(12):e0314452. doi: 10.1371/journal.pone.0314452 (PMC11651557; doi:10.1371/journal.pone.0314452)
Supplement: S1 Table — (DOCX) [file pone.0314452.s001.docx]

**S1 Table. Cancer patients’ BMI distribution.**

| **Sex** | **Tumour site** | **N** | **1%** | **5%** | **10%** | **25%** | **50%** | **75%** | **90%** | **95%** | **99%** | **Mean** | **SD** | **Min-Max** |
| --- | --- | --- | --- | --- | --- | --- | --- | --- | --- | --- | --- | --- | --- | --- |
| F | Breast | 6,962 | 17.31 | 19.03 | 20.03 | 22.04 | 24.67 | 28.04 | 31.65 | 34.29 | 39.90 | 25.45 | 4.84 | 14.20 - 57.07 |
|  | Colon | 1,517 | 16.23 | 17.95 | 19.07 | 21.22 | 24.03 | 27.69 | 31.22 | 33.70 | 38.93 | 24.77 | 4.99 | 14.69 - 54.69 |
|  | Lung | 2,168 | 15.76 | 17.78 | 18.73 | 20.94 | 23.44 | 26.56 | 29.92 | 32.82 | 38.31 | 24.09 | 4.65 | 14.01 - 58.27 |
|  | Rectum | 376 | 16.60 | 17.98 | 19.17 | 21.33 | 24.06 | 27.36 | 32.01 | 34.32 | 38.10 | 24.81 | 5.00 | 15.06 - 42.98 |
|  | Stomach | 591 | 15.63 | 17.24 | 18.26 | 20.20 | 23.05 | 26.40 | 29.72 | 32.65 | 36.93 | 23.72 | 4.80 | 14.69 - 52.44 |
|  | **Overall female** | **11,614** | **16.61** | **18.37** | **19.53** | **21.63** | **24.22** | **27.59** | **31.25** | **33.87** | **39.44** | **25.00** | **4.87** | **14.01 - 58.27** |
| M | Colon | 1,908 | 17.99 | 19.84 | 20.85 | 22.86 | 25.25 | 27.68 | 30.80 | 33.53 | 37.76 | 25.62 | 4.08 | 14.81 - 47.18 |
|  | Lung | 3,736 | 16.90 | 19.38 | 20.45 | 22.49 | 24.63 | 27.36 | 30.07 | 31.96 | 36.23 | 25.04 | 3.95 | 14.04 - 54.29 |
|  | Prostate | 1,477 | 18.59 | 20.76 | 21.80 | 23.88 | 26.12 | 29.07 | 32.25 | 34.49 | 39.24 | 26.68 | 4.22 | 15.43 - 44.19 |
|  | Rectum | 776 | 17.94 | 20.11 | 21.26 | 23.22 | 25.52 | 28.40 | 31.55 | 33.24 | 37.17 | 26.02 | 4.21 | 16.91 - 57.14 |
|  | Stomach | 1,123 | 16.77 | 18.73 | 19.88 | 21.58 | 24.02 | 26.42 | 29.35 | 31.07 | 36.71 | 24.31 | 3.92 | 13.84- 43.25 |
|  | **Overall men** | **9,020** | **17.30** | **19.53** | **20.76** | **22.68** | **24.93** | **27.68** | **30.76** | **32.87** | **37.20** | **25.42** | **4.11** | **13.84 - 57.14** |
|  | **Overall** | **20,634** | **16.85** | **18.74** | **19.95** | **22.04** | **24.61** | **27.68** | **31.14** | **33.33** | **38.72** | **25.19** | **4.55** | **13.84 - 58.27** |

The BMI (kg/m2) measures collected at the beginning of each treatment line were split according to the sex (F and M) and tumor site. Lung included both NSCLC (non-small cell lung cancer) and SCLC (small cell lung cancer). The third column (N) reports the number of new treatment lines initiated, corresponding to the number of BMI measures performed at the beginning of a new treatment. Data were expressed in the nth percentile (1%, 5%, 10%, 25%, 50%, 75%, 90%, 95% and 99%), mean, standard deviation (SD) and minimum and maximum value (Min-Max).
