# Supplementary material for: Body weight and body surface area of adult patients with selected cancers: An Italian multicenter study
Source: PLoS One. 2024 Dec 17;19(12):e0314452. doi: 10.1371/journal.pone.0314452 (PMC11651557; doi:10.1371/journal.pone.0314452)
Supplement: S2 Table — (DOCX) [file pone.0314452.s002.docx]

**S2 Table. Shapiro Wilk Normality test for BW, BSA and BMI distributions.**

|  | BW | | BSA | | BMI | |
| --- | --- | --- | --- | --- | --- | --- |
| Tumor location, Sex and treatment setting | statistic | p-value | statistic | p-value | statistic | p-value |
| *Breast F 1. Neoadjuvant* | 0.961 | <0.0001 | 0.942 | <0.0001 | 0.961 | <0.0001 |
| *Breast F 2. Adjuvant* | 0.943 | <0.0001 | 0.943 | <0.0001 | 0.943 | <0.0001 |
| *Breast F 3. Advanced* | 0.962 | <0.0001 | 0.957 | <0.0001 | 0.962 | <0.0001 |
| *Colon F 1. Neoadjuvant* | 0.952 | 0.6686 | 0.949 | 0.6289 | 0.952 | 0.6686 |
| *Colon F 2. Adjuvant* | 0.935 | <0.0001 | 0.944 | <0.0001 | 0.935 | <0.0001 |
| *Colon F 3. Advanced* | 0.957 | <0.0001 | 0.965 | <0.0001 | 0.957 | <0.0001 |
| *Colon M 1. Neoadjuvant* | 0.947 | 0.2948 | 0.927 | 0.1207 | 0.947 | 0.2948 |
| *Colon M 2. Adjuvant* | 0.966 | <0.0001 | 0.968 | <0.0001 | 0.966 | <0.0001 |
| *Colon M 3. Advanced* | 0.967 | <0.0001 | 0.956 | <0.0001 | 0.967 | <0.0001 |
| *Lung F 1. Neoadjuvant* | 0.964 | 0.0131 | 0.952 | 0.0021 | 0.964 | 0.0131 |
| *Lung F 2. Adjuvant* | 0.968 | 0.0098 | 0.957 | 0.0016 | 0.968 | 0.0098 |
| *Lung F 3. Advanced* | 0.941 | <0.0001 | 0.945 | <0.0001 | 0.941 | <0.0001 |
| *Lung M 1. Neoadjuvant* | 0.982 | 0.0119 | 0.978 | 0.0038 | 0.982 | 0.0119 |
| *Lung M 2. Adjuvant* | 0.862 | <0.0001 | 0.827 | <0.0001 | 0.862 | <0.0001 |
| *Lung M 3. Advanced* | 0.974 | <0.0001 | 0.979 | <0.0001 | 0.974 | <0.0001 |
| *Prostate M 3. Advanced* | 0.974 | <0.0001 | 0.973 | <0.0001 | 0.974 | <0.0001 |
| *Rectum F 1. Neoadjuvant* | 0.957 | 0.0008 | 0.973 | 0.0172 | 0.957 | 0.0008 |
| *Rectum F 2. Adjuvant* | 0.933 | <0.0001 | 0.949 | 0.0003 | 0.933 | <0.0001 |
| *Rectum F 3. Advanced* | 0.947 | <0.0001 | 0.950 | <0.0001 | 0.947 | <0.0001 |
| *Rectum M 1. Neoadjuvant* | 0.966 | <0.0001 | 0.956 | <0.0001 | 0.966 | <0.0001 |
| *Rectum M 2. Adjuvant* | 0.967 | 0.0001 | 0.958 | <0.0001 | 0.967 | 0.0001 |
| *Rectum M 3. Advanced* | 0.925 | <0.0001 | 0.936 | <0.0001 | 0.925 | <0.0001 |
| *Stomach F 1. Neoadjuvant* | 0.945 | 0.0011 | 0.916 | <0.0001 | 0.945 | 0.0011 |
| *Stomach F 2. Adjuvant* | 0.960 | 0.0003 | 0.976 | 0.0104 | 0.960 | 0.0003 |
| *Stomach F 3. Advanced* | 0.939 | <0.0001 | 0.919 | <0.0001 | 0.939 | <0.0001 |
| *Stomach M 1. Neoadjuvant* | 0.928 | <0.0001 | 0.956 | 0.0001 | 0.928 | <0.0001 |
| *Stomach M 2. Adjuvant* | 0.974 | 0.0003 | 0.970 | 0.0001 | 0.974 | 0.0003 |
| *Stomach M 3. Advanced* | 0.962 | <0.0001 | 0.961 | <0.0001 | 0.962 | <0.0001 |
