# Supplementary material for: Body weight and body surface area of adult patients with selected cancers: An Italian multicenter study
Source: PLoS One. 2024 Dec 17;19(12):e0314452. doi: 10.1371/journal.pone.0314452 (PMC11651557; doi:10.1371/journal.pone.0314452)
Supplement: S3 Table — (DOCX) [file pone.0314452.s003.docx]

**S3 Table. Comparison of mean cancer patients’ BSA among countries.**

| **Study** | **Overall sample**  **(%_men_;%_women_)** | **Enrollment**  **period** | **Location** | **BSA** | | |
| --- | --- | --- | --- | --- | --- | --- |
|  |  |  |  | **Men**  **(m^2^)** | **Women**  **(m^2^)** | **Overall (m^2^)** |
| Baker et al. [19] | 1650  (not reported) | 1991-2001 | USA | not reported | not reported | 1.86 |
| Dooley et al. [20] | 2838  (50.53%; 49.47%) | 1996-2000 | Australian | 1.89 | 1.70 | 1.80 |
| Sacco et al. [22] | 3613  (40.71%; 59.29%) | 2005 | UK | 1.91 | 1.71 | 1.79 |
| Goněc et al. [23] | 3873  (36.07%; 63.93%) | 2013-2014 | Czech | 2.00 | 1.78 | 1.86 |
| Current analysis | 13,036 (20,634)^a^  (43.72%; 55.28%)^b^ | 2011-2021 | Italy (Romagna) | 1.87 | 1.66 | 1.75 |

^a^ The number 20,634 represents the number of new treatment lines initiated, corresponding to the number of BSA measures performed on the sample (N=13,036).

^b^ The percentages by sex refer to the number of new treatment lines (N=20,634).
